# Crystal structure of tri­chlorido­(4′-ferrocenyl-2,2′:6′,2′′-terpyridine-κ^3^ *N*,*N*′,*N*′′)iridium(III) aceto­nitrile disolvate

**DOI:** 10.1107/S2056989015003473

**Published:** 2015-02-25

**Authors:** Bambar Davaasuren, Harihara Padhy, Alexander Rothenberger

**Affiliations:** a Physical Sciences and Engineering Division, King Abdullah University of Science and Technology, KAUST, Thuwal 23955-6900, Kingdom of , Saudi Arabia

**Keywords:** crystal structure, iridium, 4′-ferrocenyl-2,2′:6′,2′′-terpyridine

## Abstract

In the title compound, [FeIr(C_5_H_5_)(C_20_H_14_N_3_)Cl_3_]·2CH_3_CN, the central Ir^III^ atom is sixfold coordinated by three chloride ligands and three terpyridine N atoms in a slightly distorted octa­hedral fashion. The terpyridine ligand is functionalized at the 4′-position with a ferrocenyl group, the latter being in an eclipsed conformation. In the crystal, mol­ecules are stacked in rows parallel to [001], with the aceto­nitrile solvent mol­ecules situated between the rows. An extensive network of intra- and inter­molecular C—H⋯Cl inter­actions is present, stabilizing the three-dimensional structure.

## Related literature   

Complexes of metal ions with ferrocene-substituted terpyridine ligands show inter­esting electron transport dynamics (Sakamoto *et al.*, 2015 ▸), DNA cleavage and anti­cancer activity (Maity *et al.*, 2010 ▸) and enhanced electro-optical properties (Wu *et al.*, 2011 ▸). For the preparation of the ferrocenyl­ter­pyridine ligand, see: Constable *et al.* (1994 ▸).
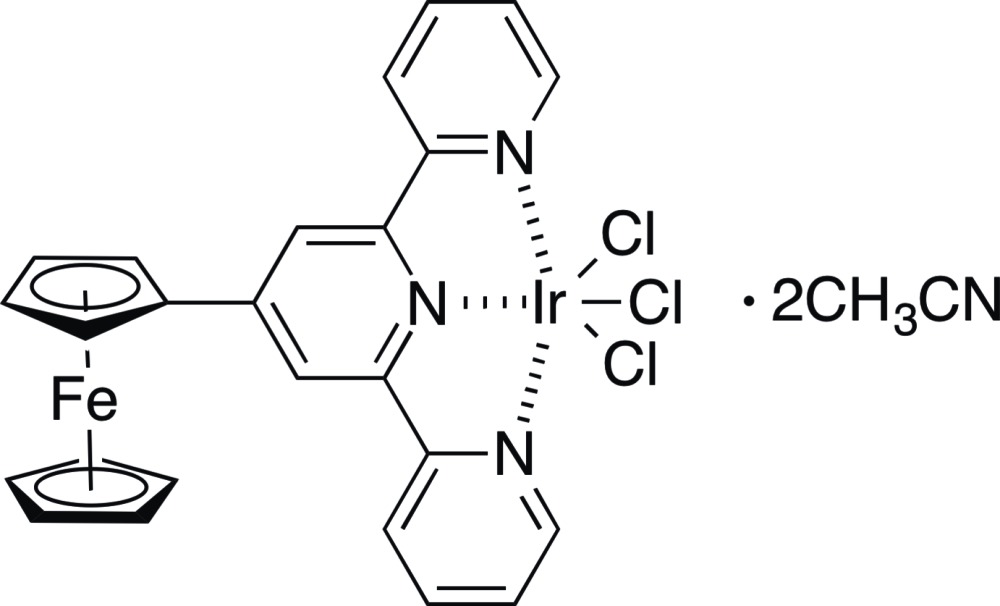


## Experimental   

### Crystal data  

[FeIr(C_5_H_5_)(C_20_H_14_N_3_)Cl_3_]·2C_2_H_3_N
*M* *_r_* = 797.94
Monoclinic, 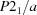

*a* = 11.557 (5) Å
*b* = 21.663 (5) Å
*c* = 11.579 (5) Å
β = 105.974 (5)°
*V* = 2787.0 (18) Å^3^
*Z* = 4
Mo *K*α radiation
μ = 5.61 mm^−1^
*T* = 150 K
0.30 × 0.23 × 0.20 mm

### Data collection  

Stoe IPDS 2 diffractometer
Absorption correction: numerical (*X-AREA* and *X-RED32*; Stoe & Cie, 2013 ▸) *T*_min_ = 0.348, *T*_max_ = 0.390
26757 measured reflections
7374 independent reflections
5312 reflections with *I* > 2σ(*I*)
*R*_int_ = 0.069

### Refinement  

*R*[*F* ^2^ > 2σ(*F* ^2^)] = 0.036
*wR*(*F* ^2^) = 0.076
*S* = 1.02
7374 reflections
354 parameters
H-atom parameters constrained
Δρ_max_ = 1.11 e Å^−3^
Δρ_min_ = −1.10 e Å^−3^

### 

Data collection: *X-AREA* (Stoe & Cie, 2013 ▸); cell refinement: *X-AREA*; data reduction: *X-AREA*; program(s) used to solve structure: *SHELXT* (Sheldrick, 2015*a* ▸); program(s) used to refine structure: *SHELXL2014* (Sheldrick, 2015*b* ▸); molecular graphics: *DIAMOND* (Brandenburg, 2006 ▸); software used to prepare material for publication: *SHELXL*.

## Supplementary Material

Crystal structure: contains datablock(s) I. DOI: 10.1107/S2056989015003473/wm5126sup1.cif

Structure factors: contains datablock(s) I. DOI: 10.1107/S2056989015003473/wm5126Isup2.hkl

**Fig. 1.**
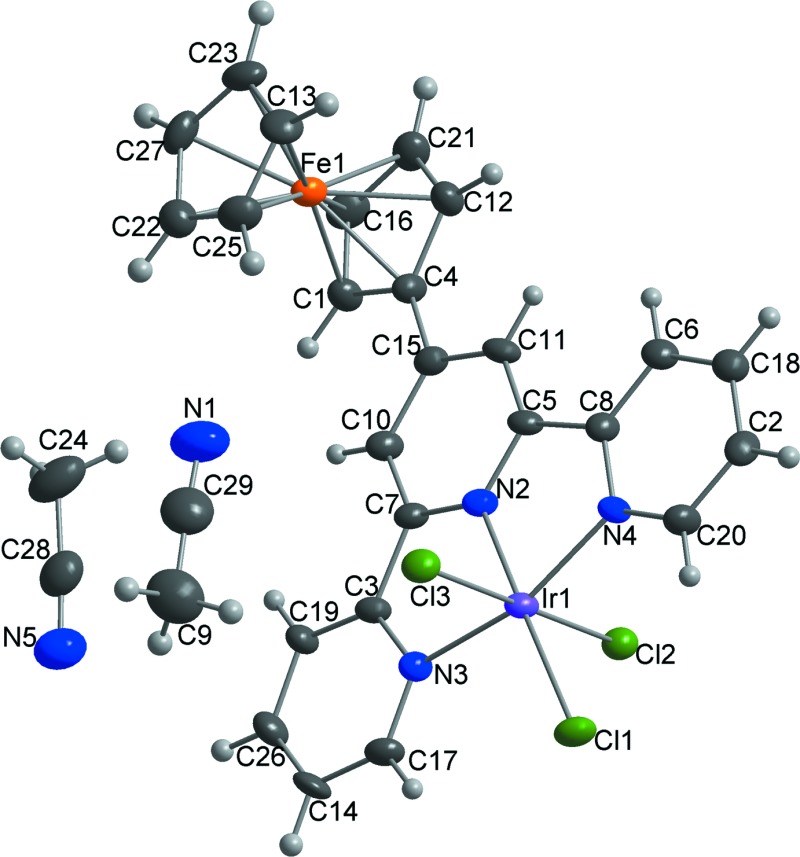
The molecular components of the title compound with displacement ellipsoids drawn at the 50% probability level. H atoms are drawn as spheres of arbitrary radius.

. DOI: 10.1107/S2056989015003473/wm5126fig1.tif
The mol­ecular components of the title compound with displacement ellipsoids drawn at the 50% probability level. H atoms are drawn as spheres of arbitrary radius.

**Fig. 2.**
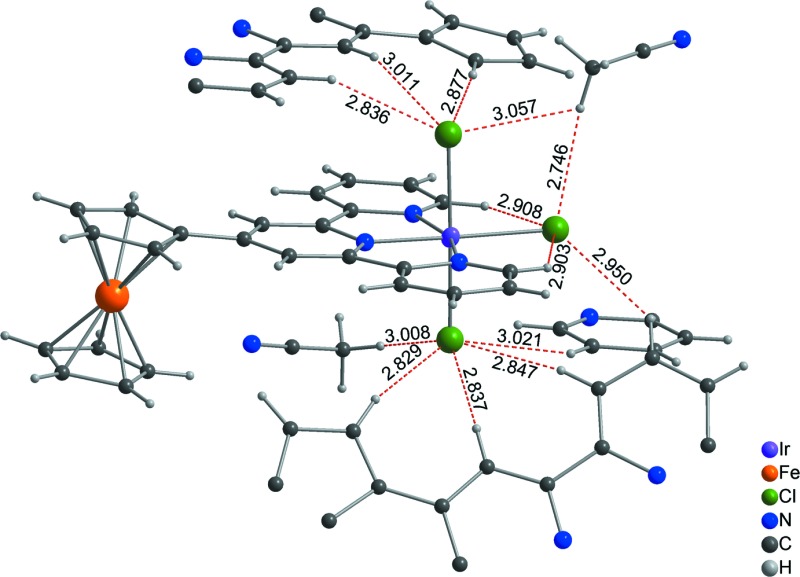
Intra- and intermolecular C—H···Cl interactions in the crystal packing, indicated by dashed-red lines.

. DOI: 10.1107/S2056989015003473/wm5126fig2.tif
Intra- and inter­molecular C—H⋯Cl inter­actions in the crystal packing, indicated by dashed-red lines.

**Fig. 3.**
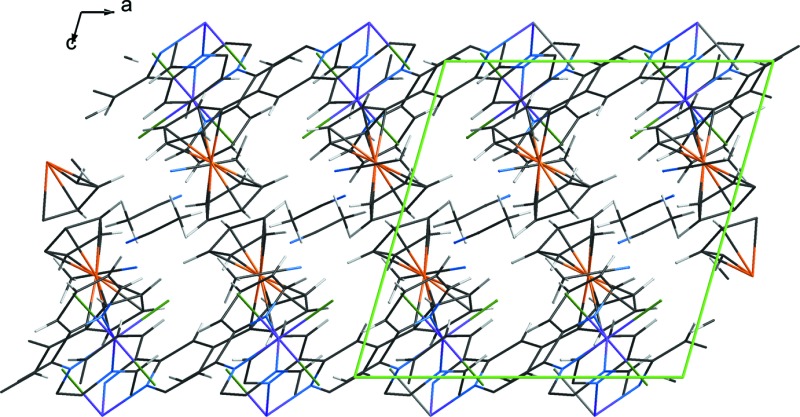
Packing diagram of the title compound viewed along [010].

. DOI: 10.1107/S2056989015003473/wm5126fig3.tif
Packing diagram of the title compound viewed along [010].

CCDC reference: 1050374

Additional supporting information:  crystallographic information; 3D view; checkCIF report

## Figures and Tables

**Table 1:** Hydrogen-bond geometry (, )

*D*H*A*	*D*H	H*A*	*D* *A*	*D*H*A*
C12H12Cl3^i^	0.98	2.83	3.771 (5)	162
C24H24*A*Cl1^ii^	0.95	2.75	3.701 (9)	174
C6H6Cl3^i^	0.95	2.83	3.731 (6)	159
C9H9*B*Cl3	0.98	2.99	3.875 (10)	151
C11H11Cl3^i^	0.95	2.82	3.711 (5)	157
C17H17Cl1	0.95	2.90	3.486 (6)	121
C20H20Cl1	0.95	2.89	3.484 (5)	122
C24H24*A*Cl1^ii^	0.98	2.73	3.701 (8)	174
C19H19Cl2^iii^	0.95	2.82	3.716 (6)	158

Symmetry codes: (i) 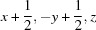; (ii) 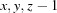; (iii) 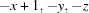.

## supplementary crystallographic information

### S1. Synthesis and crystallization

IrCl_3_ (1 mmol) was suspended in 50 ml of methanol and stirred for 20 minutes. 4'-Ferrocenyl-2,2':6',2''-terpyridine (1.2 mmol), dissolved in 3 ml THF, was dropwise added to the IrCl_3_ suspension and refluxed at 368 K overnight. The reaction mixture was cooled down to room temperature and filtered. The dark blue residue was subsequently washed with methanol and di­ethyl ether. The yield was around 80%. Rhombic dark-violet crystals were grown by diffusion from an aceto­nitrile/di­ethyl ether mixture.

### S2. Refinement

The H atoms were treated as riding and *U*_iso_(H) values set at 1.2 (aromatic) or 1.5 (methyl) *U*_eq_(C).

### Crystal data

**Table:**

[FeIr(C_5_H_5_)(C_20_H_14_N_3_)Cl_3_]·2C_2_H_3_N	*F*(000) = 1552
*M**_r_* = 797.94	*D*_x_ = 1.902 Mg m^−^^3^
Monoclinic, *P*2_1_/*a*	Mo *K*α radiation, λ = 0.71073 Å
*a* = 11.557 (5) Å	Cell parameters from 9378 reflections
*b* = 21.663 (5) Å	θ = 1.8–29.3°
*c* = 11.579 (5) Å	µ = 5.61 mm^−^^1^
β = 105.974 (5)°	*T* = 150 K
*V* = 2787.0 (18) Å^3^	Block, dark-violet
*Z* = 4	0.30 × 0.23 × 0.20 mm

### Data collection

**Table:**

STOE IPDS 2 diffractometer	7374 independent reflections
Radiation source: sealed X-ray tube, 12 x 0.4 mm long-fine focus	5312 reflections with *I* > 2σ(*I*)
Detector resolution: 6.67 pixels mm^-1^	*R*_int_ = 0.069
rotation method scans	θ_max_ = 29.2°, θ_min_ = 1.8°
Absorption correction: numerical (*X-AREA* and *X-RED32*; Stoe, 2013)	*h* = −15→15
*T*_min_ = 0.348, *T*_max_ = 0.390	*k* = −28→29
26757 measured reflections	*l* = −15→15

### Refinement

**Table:**

Refinement on *F*^2^	0 restraints
Least-squares matrix: full	Hydrogen site location: inferred from neighbouring sites
*R*[*F*^2^ > 2σ(*F*^2^)] = 0.036	H-atom parameters constrained
*wR*(*F*^2^) = 0.076	*w* = 1/[σ^2^(*F*_o_^2^) + (0.0322*P*)^2^ + 2.6254*P*] where *P* = (*F*_o_^2^ + 2*F*_c_^2^)/3
*S* = 1.02	(Δ/σ)_max_ = 0.001
7374 reflections	Δρ_max_ = 1.11 e Å^−^^3^
354 parameters	Δρ_min_ = −1.10 e Å^−^^3^

### Special details

**Table:**

Geometry. All e.s.d.'s (except the e.s.d. in the dihedral angle between two l.s. planes) are estimated using the full covariance matrix. The cell e.s.d.'s are taken into account individually in the estimation of e.s.d.'s in distances, angles and torsion angles; correlations between e.s.d.'s in cell parameters are only used when they are defined by crystal symmetry. An approximate (isotropic) treatment of cell e.s.d.'s is used for estimating e.s.d.'s involving l.s. planes.

### Fractional atomic coordinates and isotropic or equivalent isotropic displacement parameters (Å^2^)

**Table:**

	*x*	*y*	*z*	*U*_iso_*/*U*_eq_	
Ir1	0.26476 (2)	0.10206 (2)	0.12149 (2)	0.02014 (5)	
Fe1	0.62436 (7)	0.19070 (3)	−0.31981 (7)	0.02452 (15)	
Cl1	0.13455 (12)	0.07759 (6)	0.24188 (13)	0.0293 (3)	
Cl2	0.42892 (11)	0.05633 (6)	0.26314 (11)	0.0261 (2)	
Cl3	0.10318 (12)	0.14691 (6)	−0.02370 (12)	0.0277 (3)	
N1	0.2392 (8)	0.1022 (4)	−0.3477 (7)	0.083 (3)	
N2	0.3740 (4)	0.12151 (18)	0.0261 (4)	0.0213 (8)	
N3	0.2439 (4)	0.02408 (18)	0.0187 (4)	0.0229 (8)	
N4	0.3233 (4)	0.18687 (18)	0.1925 (4)	0.0217 (8)	
N5	0.1880 (7)	−0.0828 (3)	−0.4257 (6)	0.0595 (17)	
C1	0.6807 (5)	0.1118 (2)	−0.2268 (5)	0.0264 (10)	
H1	0.6479	0.0695	−0.2495	0.032*	
C2	0.3395 (5)	0.2744 (2)	0.3209 (5)	0.0282 (11)	
H2	0.3156	0.2952	0.3828	0.034*	
C3	0.3169 (4)	0.0234 (2)	−0.0569 (5)	0.0219 (9)	
C4	0.6388 (5)	0.1554 (2)	−0.1528 (5)	0.0235 (10)	
C5	0.4361 (4)	0.1751 (2)	0.0470 (5)	0.0221 (10)	
C6	0.4562 (5)	0.2697 (2)	0.1801 (5)	0.0253 (10)	
H6	0.5133	0.2875	0.1446	0.030*	
C7	0.3932 (5)	0.0789 (2)	−0.0515 (5)	0.0224 (10)	
C8	0.4053 (5)	0.2128 (2)	0.1420 (5)	0.0222 (10)	
C9	0.0512 (8)	0.0476 (4)	−0.3117 (9)	0.064 (2)	
H9A	−0.0203	0.0576	−0.3772	0.095*	
H9B	0.0399	0.0620	−0.2354	0.095*	
H9C	0.0634	0.0028	−0.3083	0.095*	
C10	0.4768 (5)	0.0895 (2)	−0.1144 (5)	0.0234 (10)	
H10	0.4892	0.0599	−0.1703	0.028*	
C11	0.5210 (5)	0.1880 (2)	−0.0131 (5)	0.0236 (10)	
H11	0.5639	0.2259	0.0006	0.028*	
C12	0.7121 (5)	0.2094 (2)	−0.1450 (5)	0.0275 (11)	
H12	0.7035	0.2482	−0.1012	0.033*	
C13	0.5157 (6)	0.2642 (2)	−0.3859 (6)	0.0341 (13)	
H13	0.5050	0.3018	−0.3399	0.041*	
C14	0.1662 (5)	−0.0745 (2)	−0.0555 (5)	0.0313 (12)	
H14	0.1132	−0.1079	−0.0543	0.038*	
C15	0.5438 (5)	0.1451 (2)	−0.0944 (5)	0.0228 (10)	
C16	0.7782 (5)	0.1396 (3)	−0.2599 (5)	0.0320 (12)	
H16	0.8245	0.1203	−0.3119	0.038*	
C17	0.1695 (5)	−0.0228 (2)	0.0186 (5)	0.0253 (10)	
H17	0.1177	−0.0213	0.0696	0.030*	
C18	0.4226 (5)	0.3002 (2)	0.2706 (5)	0.0277 (11)	
H18	0.4571	0.3392	0.2980	0.033*	
C19	0.3176 (5)	−0.0262 (2)	−0.1311 (5)	0.0252 (10)	
H19	0.3692	−0.0265	−0.1824	0.030*	
C20	0.2910 (5)	0.2170 (2)	0.2793 (5)	0.0267 (10)	
H20	0.2334	0.1988	0.3137	0.032*	
C21	0.7980 (5)	0.1994 (3)	−0.2092 (5)	0.0332 (12)	
H21	0.8599	0.2297	−0.2192	0.040*	
C22	0.4898 (6)	0.1674 (3)	−0.4711 (5)	0.0347 (13)	
H22	0.4583	0.1249	−0.4947	0.042*	
C23	0.6027 (6)	0.2562 (3)	−0.4513 (6)	0.0365 (13)	
H23	0.6641	0.2872	−0.4594	0.044*	
C24	0.3273 (8)	−0.0102 (4)	−0.5027 (7)	0.058 (2)	
H24A	0.2789	0.0110	−0.5746	0.088*	
H24B	0.3902	−0.0346	−0.5233	0.088*	
H24C	0.3646	0.0204	−0.4414	0.088*	
C25	0.4470 (5)	0.2092 (3)	−0.3980 (6)	0.0348 (13)	
H25	0.3794	0.2013	−0.3614	0.042*	
C26	0.2412 (5)	−0.0762 (2)	−0.1299 (6)	0.0291 (11)	
H26	0.2409	−0.1110	−0.1799	0.035*	
C27	0.5857 (6)	0.1966 (3)	−0.5037 (5)	0.0358 (13)	
H27	0.6343	0.1780	−0.5542	0.043*	
C28	0.2512 (7)	−0.0504 (3)	−0.4568 (6)	0.0462 (16)	
C29	0.1552 (8)	0.0774 (4)	−0.3329 (8)	0.057 (2)	

### Atomic displacement parameters (Å^2^)

**Table:**

	*U*^11^	*U*^22^	*U*^33^	*U*^12^	*U*^13^	*U*^23^
Ir1	0.02160 (8)	0.01507 (7)	0.02675 (8)	0.00012 (8)	0.01168 (6)	0.00098 (8)
Fe1	0.0263 (4)	0.0222 (3)	0.0289 (4)	0.0001 (3)	0.0140 (3)	0.0010 (3)
Cl1	0.0290 (6)	0.0285 (6)	0.0366 (7)	0.0003 (5)	0.0193 (6)	0.0053 (5)
Cl2	0.0270 (6)	0.0230 (5)	0.0295 (6)	0.0024 (4)	0.0099 (5)	0.0016 (4)
Cl3	0.0282 (6)	0.0231 (5)	0.0330 (6)	0.0048 (5)	0.0102 (5)	0.0033 (5)
N1	0.089 (6)	0.081 (5)	0.073 (5)	−0.031 (5)	0.013 (4)	0.022 (5)
N2	0.022 (2)	0.0174 (17)	0.026 (2)	0.0003 (14)	0.0098 (17)	0.0027 (15)
N3	0.026 (2)	0.0193 (18)	0.026 (2)	−0.0014 (16)	0.0117 (18)	−0.0014 (16)
N4	0.025 (2)	0.0174 (18)	0.026 (2)	−0.0037 (15)	0.0130 (17)	−0.0025 (15)
N5	0.074 (5)	0.048 (3)	0.060 (4)	−0.007 (3)	0.026 (4)	0.010 (3)
C1	0.028 (2)	0.025 (3)	0.030 (2)	0.0002 (19)	0.013 (2)	−0.0027 (19)
C2	0.039 (3)	0.019 (2)	0.028 (3)	0.007 (2)	0.013 (2)	0.0005 (19)
C3	0.022 (2)	0.017 (2)	0.028 (2)	0.0016 (17)	0.010 (2)	0.0019 (18)
C4	0.028 (2)	0.020 (2)	0.026 (2)	0.0006 (19)	0.014 (2)	−0.0013 (18)
C5	0.024 (2)	0.0145 (19)	0.029 (2)	−0.0016 (17)	0.010 (2)	0.0037 (17)
C6	0.029 (3)	0.016 (2)	0.032 (3)	0.0013 (18)	0.012 (2)	0.0012 (19)
C7	0.024 (2)	0.019 (2)	0.027 (2)	0.0020 (18)	0.012 (2)	0.0027 (18)
C8	0.026 (2)	0.0145 (19)	0.027 (2)	−0.0001 (17)	0.009 (2)	0.0007 (17)
C9	0.051 (4)	0.057 (5)	0.082 (6)	0.009 (4)	0.016 (4)	0.002 (4)
C10	0.027 (2)	0.019 (2)	0.028 (2)	0.0011 (17)	0.012 (2)	−0.0010 (17)
C11	0.023 (2)	0.018 (2)	0.031 (3)	−0.0020 (18)	0.009 (2)	0.0004 (19)
C12	0.027 (3)	0.026 (2)	0.031 (3)	−0.0046 (19)	0.010 (2)	−0.004 (2)
C13	0.042 (3)	0.019 (2)	0.042 (3)	0.007 (2)	0.013 (3)	0.005 (2)
C14	0.036 (3)	0.019 (2)	0.038 (3)	−0.014 (2)	0.008 (3)	−0.004 (2)
C15	0.026 (2)	0.020 (2)	0.025 (2)	0.0009 (18)	0.011 (2)	0.0032 (18)
C16	0.029 (3)	0.037 (3)	0.036 (3)	0.006 (2)	0.018 (2)	0.002 (2)
C17	0.023 (2)	0.022 (2)	0.033 (3)	−0.0022 (19)	0.012 (2)	0.005 (2)
C18	0.031 (3)	0.021 (2)	0.031 (3)	0.0035 (19)	0.009 (2)	0.0012 (19)
C19	0.028 (3)	0.020 (2)	0.030 (3)	−0.0042 (19)	0.010 (2)	−0.0033 (19)
C20	0.029 (3)	0.024 (2)	0.032 (3)	0.000 (2)	0.016 (2)	0.000 (2)
C21	0.027 (3)	0.042 (3)	0.033 (3)	−0.003 (2)	0.012 (2)	−0.002 (2)
C22	0.038 (3)	0.031 (3)	0.034 (3)	0.001 (2)	0.006 (3)	0.000 (2)
C23	0.037 (3)	0.034 (3)	0.039 (3)	−0.002 (2)	0.011 (3)	0.015 (2)
C24	0.058 (5)	0.067 (5)	0.054 (4)	0.008 (4)	0.023 (4)	0.025 (4)
C25	0.030 (3)	0.036 (3)	0.041 (3)	0.001 (2)	0.015 (3)	0.006 (2)
C26	0.032 (3)	0.020 (2)	0.034 (3)	−0.004 (2)	0.007 (2)	−0.005 (2)
C27	0.043 (3)	0.044 (3)	0.024 (3)	0.010 (3)	0.014 (3)	0.005 (2)
C28	0.055 (4)	0.049 (4)	0.036 (3)	0.007 (3)	0.013 (3)	0.004 (3)
C29	0.060 (5)	0.042 (4)	0.069 (5)	0.009 (4)	0.018 (4)	0.005 (4)

### Geometric parameters (Å, º)

**Table:**

Ir1—N2	1.939 (4)	C6—H6	0.9500
Ir1—N3	2.042 (4)	C7—C10	1.379 (7)
Ir1—N4	2.050 (4)	C9—C29	1.444 (12)
Ir1—Cl3	2.3516 (14)	C9—H9A	0.9800
Ir1—Cl2	2.3575 (14)	C9—H9B	0.9800
Ir1—Cl1	2.3756 (15)	C9—H9C	0.9800
Fe1—C1	2.029 (5)	C10—C15	1.416 (7)
Fe1—C12	2.040 (5)	C10—H10	0.9500
Fe1—C25	2.040 (6)	C11—C15	1.398 (7)
Fe1—C13	2.041 (5)	C11—H11	0.9500
Fe1—C4	2.044 (5)	C12—C21	1.411 (8)
Fe1—C23	2.046 (6)	C12—H12	1.0000
Fe1—C16	2.047 (6)	C13—C25	1.416 (8)
Fe1—C27	2.056 (6)	C13—C23	1.426 (9)
Fe1—C22	2.059 (6)	C13—H13	1.0000
Fe1—C21	2.071 (6)	C14—C26	1.380 (9)
N1—C29	1.163 (11)	C14—C17	1.405 (7)
N2—C7	1.349 (6)	C14—H14	0.9500
N2—C5	1.351 (6)	C16—C21	1.416 (8)
N3—C17	1.331 (6)	C16—H16	1.0000
N3—C3	1.372 (7)	C17—H17	0.9500
N4—C20	1.334 (7)	C18—H18	0.9500
N4—C8	1.364 (6)	C19—C26	1.400 (7)
N5—C28	1.142 (10)	C19—H19	0.9500
C1—C16	1.420 (8)	C20—H20	0.9500
C1—C4	1.445 (7)	C21—H21	1.0000
C1—H1	1.0000	C22—C27	1.415 (9)
C2—C18	1.372 (8)	C22—C25	1.419 (8)
C2—C20	1.395 (7)	C22—H22	1.0000
C2—H2	0.9500	C23—C27	1.417 (9)
C3—C19	1.377 (7)	C23—H23	1.0000
C3—C7	1.483 (7)	C24—C28	1.438 (11)
C4—C12	1.433 (7)	C24—H24A	0.9800
C4—C15	1.457 (7)	C24—H24B	0.9800
C5—C11	1.379 (7)	C24—H24C	0.9800
C5—C8	1.489 (7)	C25—H25	1.0000
C6—C18	1.382 (8)	C26—H26	0.9500
C6—C8	1.386 (7)	C27—H27	1.0000
			
N2—Ir1—N3	80.72 (17)	N2—C7—C3	112.2 (4)
N2—Ir1—N4	80.75 (17)	C10—C7—C3	127.4 (5)
N3—Ir1—N4	161.46 (17)	N4—C8—C6	120.9 (5)
N2—Ir1—Cl3	90.93 (13)	N4—C8—C5	115.3 (4)
N3—Ir1—Cl3	88.97 (13)	C6—C8—C5	123.8 (5)
N4—Ir1—Cl3	91.49 (12)	C29—C9—H9A	109.5
N2—Ir1—Cl2	87.84 (13)	C29—C9—H9B	109.5
N3—Ir1—Cl2	90.07 (13)	H9A—C9—H9B	109.5
N4—Ir1—Cl2	89.08 (12)	C29—C9—H9C	109.5
Cl3—Ir1—Cl2	178.55 (5)	H9A—C9—H9C	109.5
N2—Ir1—Cl1	178.77 (13)	H9B—C9—H9C	109.5
N3—Ir1—Cl1	99.50 (13)	C7—C10—C15	119.1 (5)
N4—Ir1—Cl1	99.03 (12)	C7—C10—H10	120.5
Cl3—Ir1—Cl1	90.29 (6)	C15—C10—H10	120.5
Cl2—Ir1—Cl1	90.94 (6)	C5—C11—C15	119.8 (4)
C1—Fe1—C12	69.0 (2)	C5—C11—H11	120.1
C1—Fe1—C25	122.5 (2)	C15—C11—H11	120.1
C12—Fe1—C25	124.4 (2)	C21—C12—C4	109.3 (5)
C1—Fe1—C13	158.4 (2)	C21—C12—Fe1	71.1 (3)
C12—Fe1—C13	107.7 (2)	C4—C12—Fe1	69.6 (3)
C25—Fe1—C13	40.6 (2)	C21—C12—H12	125.3
C1—Fe1—C4	41.56 (19)	C4—C12—H12	125.3
C12—Fe1—C4	41.1 (2)	Fe1—C12—H12	125.3
C25—Fe1—C4	107.7 (2)	C25—C13—C23	107.7 (5)
C13—Fe1—C4	121.9 (2)	C25—C13—Fe1	69.7 (3)
C1—Fe1—C23	159.2 (2)	C23—C13—Fe1	69.8 (3)
C12—Fe1—C23	121.7 (2)	C25—C13—H13	126.1
C25—Fe1—C23	68.3 (2)	C23—C13—H13	126.1
C13—Fe1—C23	40.8 (3)	Fe1—C13—H13	126.1
C4—Fe1—C23	157.7 (2)	C26—C14—C17	119.1 (5)
C1—Fe1—C16	40.8 (2)	C26—C14—H14	120.5
C12—Fe1—C16	67.9 (2)	C17—C14—H14	120.5
C25—Fe1—C16	158.4 (2)	C11—C15—C10	118.7 (5)
C13—Fe1—C16	159.4 (2)	C11—C15—C4	121.1 (4)
C4—Fe1—C16	68.9 (2)	C10—C15—C4	120.2 (4)
C23—Fe1—C16	123.0 (3)	C21—C16—C1	109.0 (5)
C1—Fe1—C27	123.3 (2)	C21—C16—Fe1	70.8 (3)
C12—Fe1—C27	157.3 (2)	C1—C16—Fe1	68.9 (3)
C25—Fe1—C27	67.8 (3)	C21—C16—H16	125.5
C13—Fe1—C27	68.1 (2)	C1—C16—H16	125.5
C4—Fe1—C27	160.3 (2)	Fe1—C16—H16	125.5
C23—Fe1—C27	40.4 (3)	N3—C17—C14	121.2 (5)
C16—Fe1—C27	107.8 (2)	N3—C17—H17	119.4
C1—Fe1—C22	107.6 (2)	C14—C17—H17	119.4
C12—Fe1—C22	161.0 (2)	C2—C18—C6	120.2 (5)
C25—Fe1—C22	40.5 (2)	C2—C18—H18	119.9
C13—Fe1—C22	68.2 (2)	C6—C18—H18	119.9
C4—Fe1—C22	124.0 (2)	C3—C19—C26	119.0 (5)
C23—Fe1—C22	68.1 (2)	C3—C19—H19	120.5
C16—Fe1—C22	122.6 (2)	C26—C19—H19	120.5
C27—Fe1—C22	40.2 (3)	N4—C20—C2	121.8 (5)
C1—Fe1—C21	68.5 (2)	N4—C20—H20	119.1
C12—Fe1—C21	40.1 (2)	C2—C20—H20	119.1
C25—Fe1—C21	160.2 (2)	C12—C21—C16	107.6 (5)
C13—Fe1—C21	123.5 (2)	C12—C21—Fe1	68.7 (3)
C4—Fe1—C21	68.6 (2)	C16—C21—Fe1	69.0 (3)
C23—Fe1—C21	107.2 (2)	C12—C21—H21	126.2
C16—Fe1—C21	40.2 (2)	C16—C21—H21	126.2
C27—Fe1—C21	122.2 (3)	Fe1—C21—H21	126.2
C22—Fe1—C21	157.6 (2)	C27—C22—C25	107.6 (5)
C7—N2—C5	122.1 (4)	C27—C22—Fe1	69.8 (3)
C7—N2—Ir1	118.9 (3)	C25—C22—Fe1	69.0 (3)
C5—N2—Ir1	118.8 (3)	C27—C22—H22	126.2
C17—N3—C3	120.0 (4)	C25—C22—H22	126.2
C17—N3—Ir1	127.2 (4)	Fe1—C22—H22	126.2
C3—N3—Ir1	112.7 (3)	C27—C23—C13	107.6 (5)
C20—N4—C8	119.6 (4)	C27—C23—Fe1	70.2 (3)
C20—N4—Ir1	127.6 (3)	C13—C23—Fe1	69.4 (3)
C8—N4—Ir1	112.8 (3)	C27—C23—H23	126.2
C16—C1—C4	107.7 (5)	C13—C23—H23	126.2
C16—C1—Fe1	70.3 (3)	Fe1—C23—H23	126.2
C4—C1—Fe1	69.8 (3)	C28—C24—H24A	109.5
C16—C1—H1	126.1	C28—C24—H24B	109.5
C4—C1—H1	126.1	H24A—C24—H24B	109.5
Fe1—C1—H1	126.1	C28—C24—H24C	109.5
C18—C2—C20	118.6 (5)	H24A—C24—H24C	109.5
C18—C2—H2	120.7	H24B—C24—H24C	109.5
C20—C2—H2	120.7	C13—C25—C22	108.5 (5)
N3—C3—C19	121.2 (4)	C13—C25—Fe1	69.7 (3)
N3—C3—C7	115.3 (4)	C22—C25—Fe1	70.5 (3)
C19—C3—C7	123.5 (5)	C13—C25—H25	125.8
C12—C4—C1	106.4 (5)	C22—C25—H25	125.8
C12—C4—C15	127.2 (5)	Fe1—C25—H25	125.8
C1—C4—C15	126.4 (4)	C14—C26—C19	119.5 (5)
C12—C4—Fe1	69.3 (3)	C14—C26—H26	120.3
C1—C4—Fe1	68.7 (3)	C19—C26—H26	120.3
C15—C4—Fe1	128.4 (4)	C22—C27—C23	108.6 (5)
N2—C5—C11	120.0 (5)	C22—C27—Fe1	70.0 (3)
N2—C5—C8	112.3 (4)	C23—C27—Fe1	69.4 (3)
C11—C5—C8	127.6 (4)	C22—C27—H27	125.7
C18—C6—C8	119.0 (5)	C23—C27—H27	125.7
C18—C6—H6	120.5	Fe1—C27—H27	125.7
C8—C6—H6	120.5	N5—C28—C24	176.8 (8)
N2—C7—C10	120.4 (4)	N1—C29—C9	178.6 (11)

### Hydrogen-bond geometry (Å, º)

**Table:**

*D*—H···*A*	*D*—H	H···*A*	*D*···*A*	*D*—H···*A*
C12—H12···Cl3^i^	0.98	2.83	3.771 (5)	162
C24—H24*A*···Cl1^ii^	0.95	2.75	3.701 (9)	174
C6—H6···Cl3^i^	0.95	2.83	3.731 (6)	159
C9—H9*B*···Cl3	0.98	2.99	3.875 (10)	151
C11—H11···Cl3^i^	0.95	2.82	3.711 (5)	157
C17—H17···Cl1	0.95	2.90	3.486 (6)	121
C20—H20···Cl1	0.95	2.89	3.484 (5)	122
C24—H24*A*···Cl1^ii^	0.98	2.73	3.701 (8)	174
C19—H19···Cl2^iii^	0.95	2.82	3.716 (6)	158

Symmetry codes: (i) *x*+1/2, −*y*+1/2, *z*; (ii) *x*, *y*, *z*−1; (iii) −*x*+1, −*y*, −*z*.
