# Supplementary figures and images for: Crystal structure of tri­chlorido­(4′-ferrocenyl-2,2′:6′,2′′-terpyridine-κ3 N,N′,N′′)iridium(III) aceto­nitrile disolvate
Source: Acta Crystallogr E Crystallogr Commun. 2015 Feb 25;71(Pt 3):m69–70. doi: 10.1107/S2056989015003473 (PMC4350693; doi:10.1107/S2056989015003473)

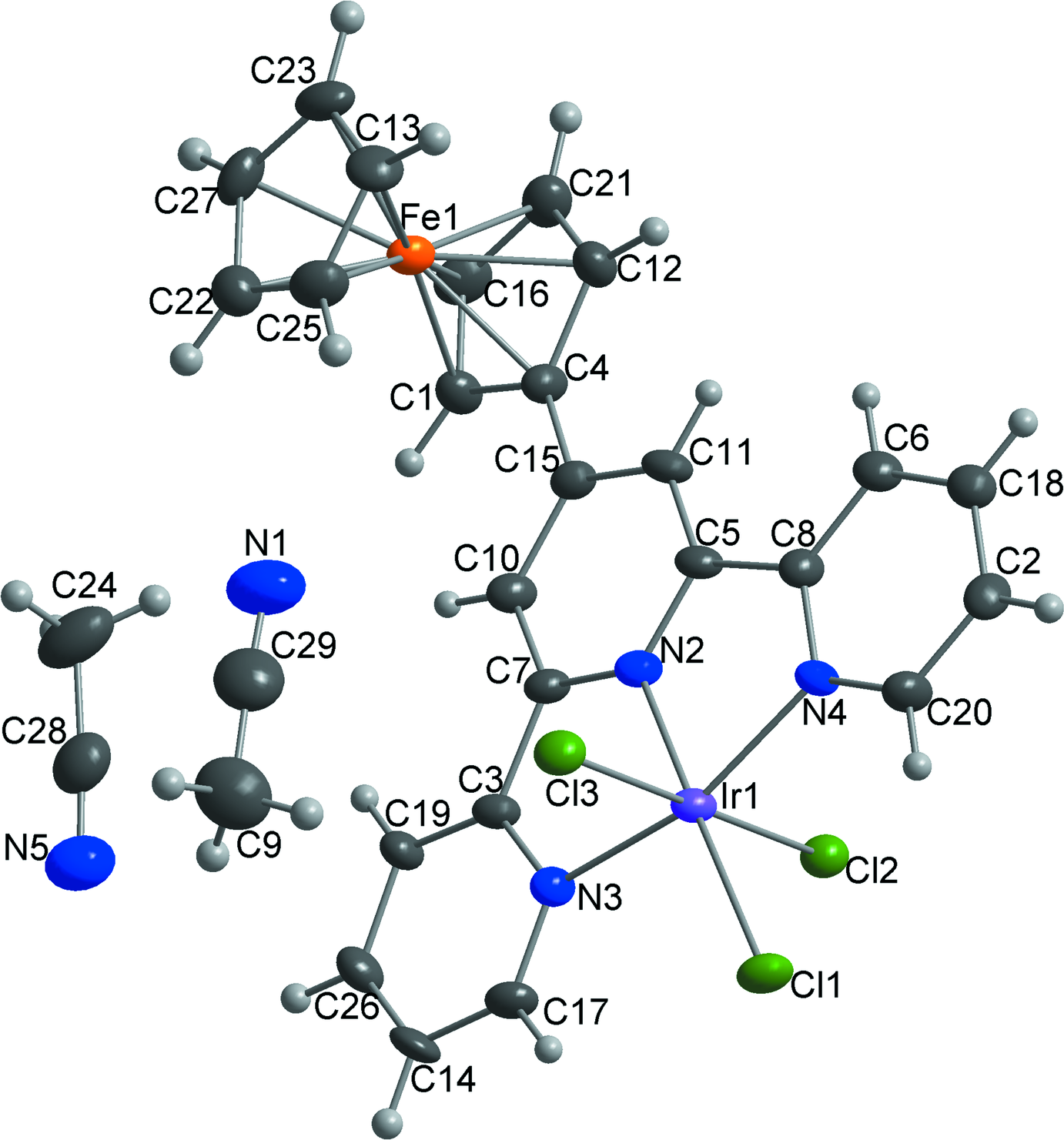

Supplement: Supplementary file 3 [file e-71-00m69-fig1.tif]

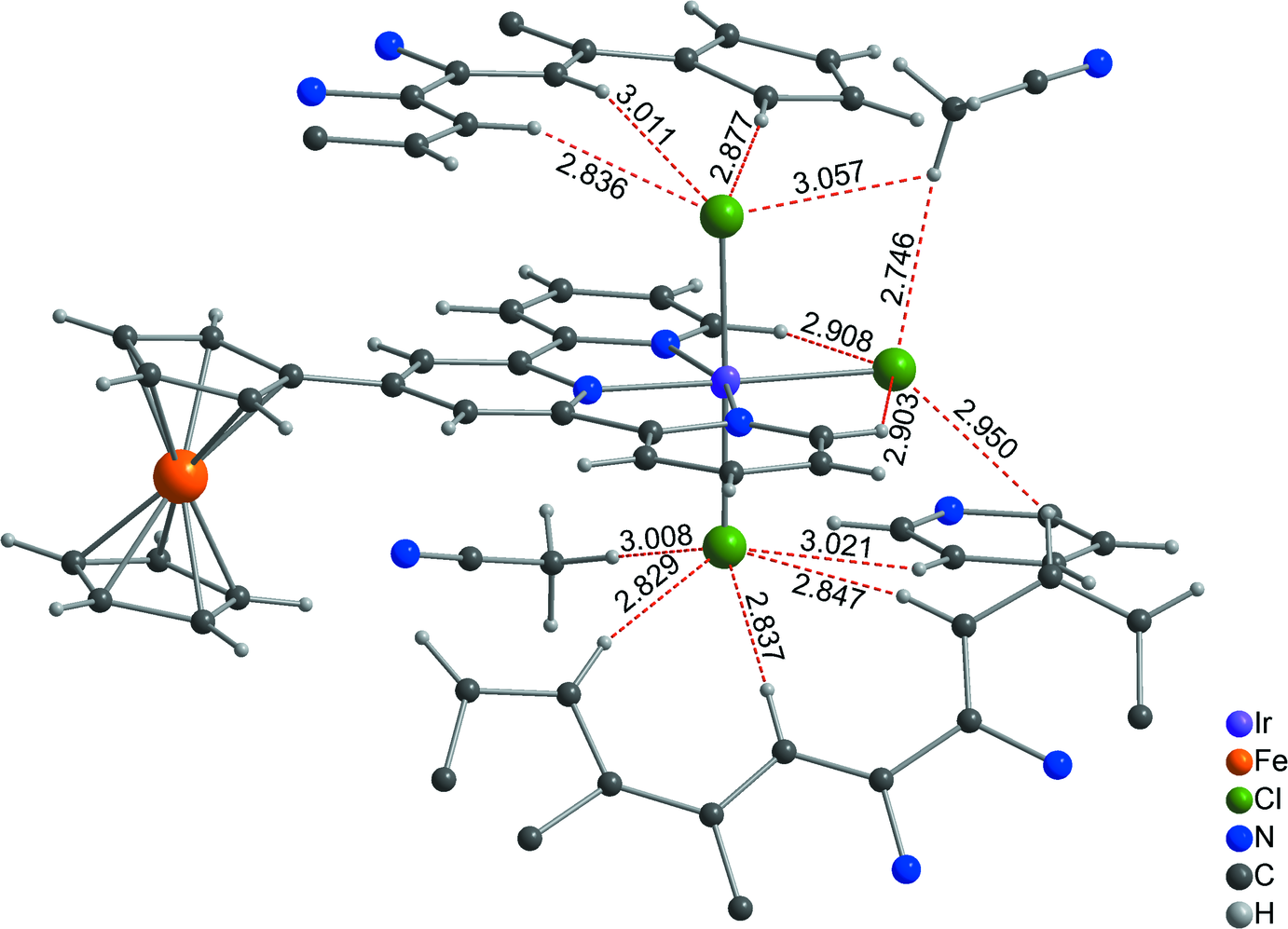

Supplement: Supplementary file 4 [file e-71-00m69-fig2.tif]

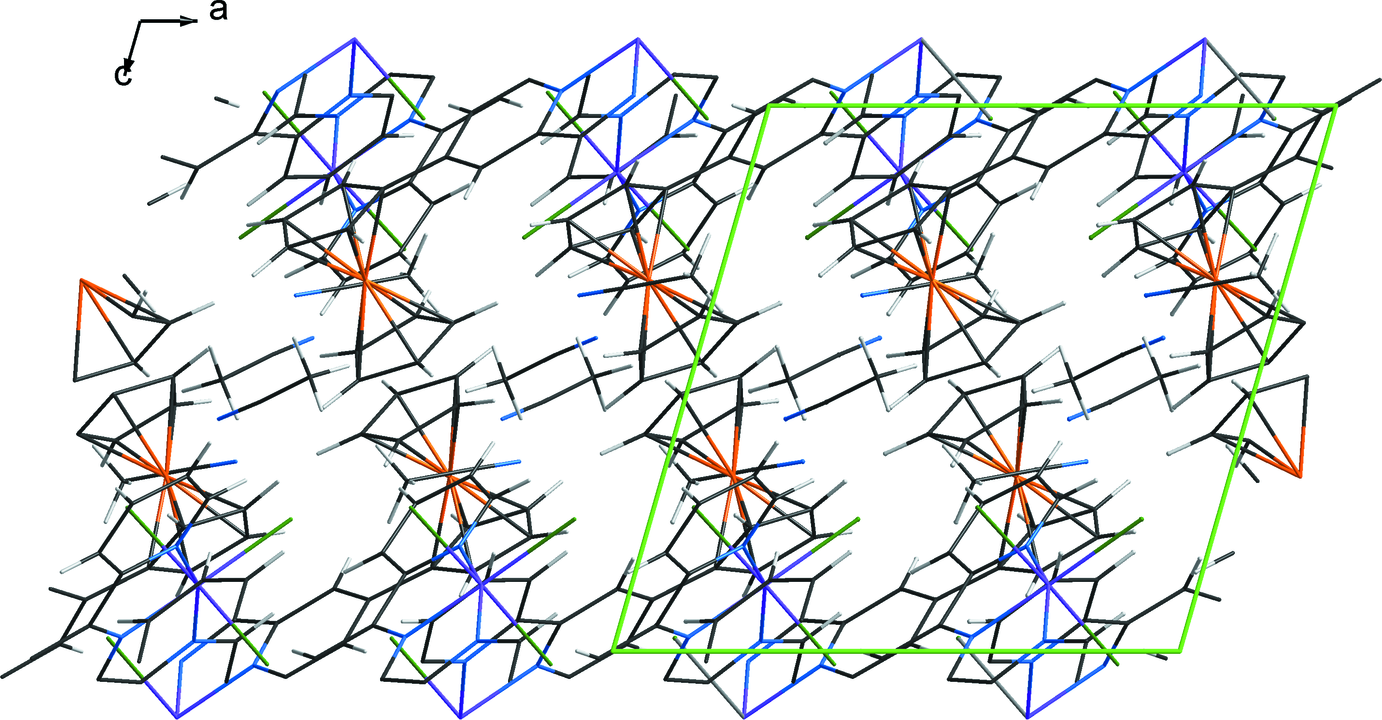

Supplement: Supplementary file 5 [file e-71-00m69-fig3.tif]
